# GDF15 and Cardiac Cells: Current Concepts and New Insights

**DOI:** 10.3390/ijms22168889

**Published:** 2021-08-18

**Authors:** Luc Rochette, Geoffrey Dogon, Marianne Zeller, Yves Cottin, Catherine Vergely

**Affiliations:** 1 Pathophysiology and Epidemiology of Cerebro-Cardiovascular Disease Research Unit (PEC2, EA 7460), Faculty of Health Sciences (UFR des Sciences de Santé), University of Burgundy and Franche-Comté, 7 Boulevard Jeanne d’Arc, 21079 Dijon, France; geoffrey.dogon@u-bourgogne.fr (G.D.); marianne.zeller@u-bourgogne.fr (M.Z.); cvergely@u-bourgogne.fr (C.V.); 2 Cardiology Unit, CHU Dijon Bourgogne, 21000 Dijon, France; yves.cottin@chu-dijon.fr

**Keywords:** GDF15, cardiac hormone, biomarker, cardiovascular disease

## Abstract

Growth and differentiation factor 15 (GDF15) belongs to the transforming growth factor-β (TGF-β) superfamily of proteins. Glial-derived neurotrophic factor (GDNF) family receptor α-like (GFRAL) is an endogenous receptor for GDF15 detected selectively in the brain. GDF15 is not normally expressed in the tissue but is prominently induced by “injury”. Serum levels of GDF15 are also increased by aging and in response to cellular stress and mitochondrial dysfunction. It acts as an inflammatory marker and plays a role in the pathogenesis of cardiovascular diseases, metabolic disorders, and neurodegenerative processes. Identified as a new heart-derived endocrine hormone that regulates body growth, GDF15 has a local cardioprotective role, presumably due to its autocrine/paracrine properties: antioxidative, anti-inflammatory, antiapoptotic. GDF15 expression is highly induced in cardiomyocytes after ischemia/reperfusion and in the heart within hours after myocardial infarction (MI). Recent studies show associations between GDF15, inflammation, and cardiac fibrosis during heart failure and MI. However, the reason for this increase in GDF15 production has not been clearly identified. Experimental and clinical studies support the potential use of GDF15 as a novel therapeutic target (1) by modulating metabolic activity and (2) promoting an adaptive angiogenesis and cardiac regenerative process during cardiovascular diseases. In this review, we comment on new aspects of the biology of GDF15 as a cardiac hormone and show that GDF15 may be a predictive biomarker of adverse cardiac events.

## 1. Introduction

Cardiomyogenesis during mammalian heart development is initiated with the involvement of cardiogenic precursor cells derived from the embryonic mesoderm, which themselves are specified through a series of paracrine and autocrine gene regulatory signals. These cardiac progenitors undergo rapid proliferation and differentiation to generate and expand the early heart tube [1]. Cardiogenesis is regulated by multiple cell types, including cardiomyocytes, fibroblasts, endothelial cells (ECs), smooth muscle cells (SMC), and hematopoietic-derived cells. ECs constitute the majority of non-cardiomyocytes in the heart and are involved in multiple regulatory and disease responses in the myocardium. The human heart contains an estimated 2–3 billion cardiac muscle cells. In vivo adult cardiomyocytes have a large, anisotropic, rod-like shape that is approximately 150 μm in length, 20 μm in width, 15 μm in height and 40,000 μm^3^ in volume [2]. It has been reported that the cellular composition of the heart is heterogeneous, plastic, and sex-specific, with differences between atrial and ventricular tissue. Atrial tissue contains 30.1% cardiomyocytes, 24.3% fibroblasts, 17.1% mural cells (pericytes and vascular smooth muscle cells), 12.2% endothelial cells, and 10.4% immune cells (myeloid and lymphoid cells). Ventricular tissue, however, contains 49.2% cardiomyocytes, 21.2% mural cells, 15.5% fibroblasts, 7.8% endothelial cells, and 5.3% immune cells. Sex-specific differences in the composition of hearts develop shortly after birth under the control of gonadal hormones [3,4].

During the first days of postnatal life, the mammalian heart undergoes substantial growth and physiological remodeling to accommodate an increased demand for cardiac output. After birth, the developmental changes in cardiomyocytes are significant. They grow massively, with a 10 to 20-fold increase in cell volume. Myocardial growth in humans is based on two cellular mechanisms: cardiomyocyte enlargement and proliferation. During heart development, the presence of fibroblasts, ECs, SMC, neurons, and immune cells contributes to cardiomyocyte maturation. Cardiac fibroblasts have specific roles during heart development. Initially, embryonic fibroblasts promote the proliferation of cardiomyocytes by secretion of extracellular matrix (ECM) factors, while adult fibroblasts participate in cardiomyocyte organization by secreting growth cytokines. Additional factors produced locally in the heart or delivered via the circulation, such as insulin-like growth factor 1 (IGF1) and neuregulin 1β (NRG1), promote physiological cardiomyocyte hypertrophy [5]. NRG1 and its signaling receptors, erythroblastic leukemia viral oncogene homologs (ErbB) 2, 3, and 4, have been implicated in both cardiomyocyte development and in homeostatic cardiac function. ErbB2 is a member of the epidermal growth factor receptor (EGFR) family. NRG-1/ErbB signaling is involved in a multitude of cardiac processes ranging from the angiogenic support of cardiomyocytes to protection upon injury [6].

The ECM plays a crucial role in cardiac homeostasis, not only by providing structural support but also by facilitating force transmission and transducing key signals to cardiomyocytes, vascular cells, and interstitial cells [7]. During myocardial development, the distinct cell pools are not isolated from one another but instead interact physically and via a variety of soluble paracrine, autocrine, and endocrine factors. Cardiomyocyte–cardiomyocyte communication occurs through several channels, including the secretion of autocrine factors, and physical association via gap junctions and adhesion complexes [8]. The intercalated disc is the cell–cell junction that longitudinally connects neighboring cardiac myocytes. The intercalated disc has multiple functions related to the maintenance of mechanical and electrical coupling between cardiomyocytes. The three types of cell junctions that make up intercalated discs are fascia adherens, desmosomes, and gap junctions [9]. Normal cardiac myocardium shows a laminar organization of myocardial “sheet-like” structures enclosed around the left ventricle and bridged by small trabeculae at regular intervals [10]. Intercellular communications play a pivotal role through subtypes of extracellular vesicles, so-called exosomes, known as important intercellular communication mediators in the heart. After myocardial infarction (MI), exosomes, especially those secreted by cardiac stem cells, have been shown to confer cardioprotective effects. They activate regenerative signals, participating in cardiac repair. The molecular content of exosomes depends on the cell type and the functional state of the producing cell. Their biological cargo includes lipids, proteins, microRNAs (miRNAs), and long noncoding RNAs (lncRNAs). Most studies have focused on the function of different miRNAs in exosomes [11,12].

**Figure 1:**
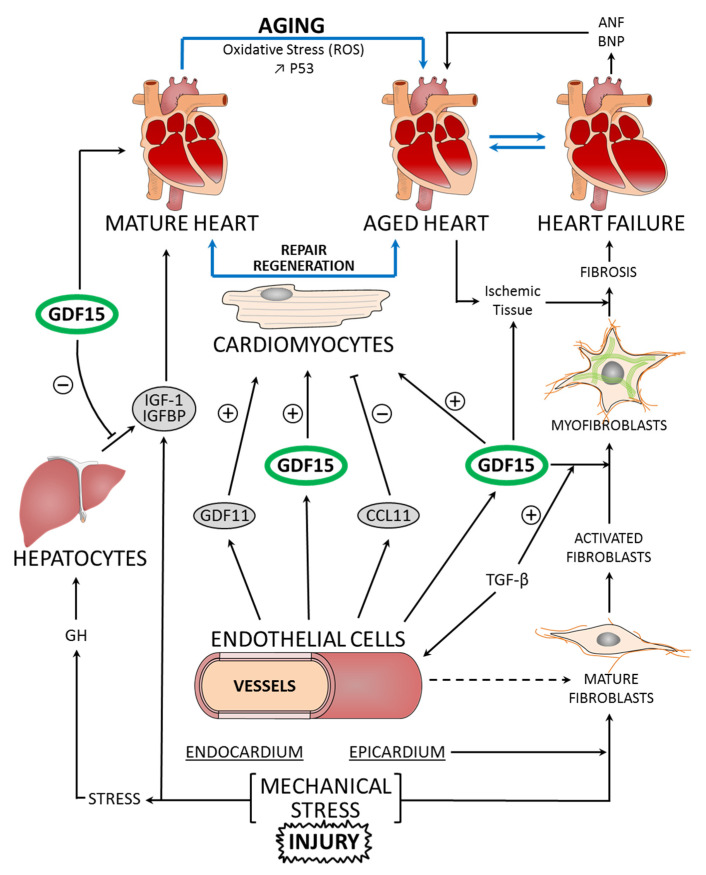
Molecular pathways linking mature heart, aged heart, and heart failure to GDF15 function, in the regulation of cardiomyocyte repair and regeneration. Aging is characterized by increased oxidative stress through reactive oxygen species (ROS) production and DNA damage. The mechanical stress and injury on the myocardium induce the secretion and release of GDF15. GDF15 shows a cardioprotective effect on the cardiomyocytes of ischemic tissue. Other endogenous factors, such as GDF11 and CCL11, also influence the regeneration process. The development of fibrosis is correlated with heart failure. The transformation of cardiac fibroblasts to myofibroblasts is a process in the adaptation of the myocardium to stress. GDF15 and TGF-β are factors controlling this transformation. In heart failure, ANP and BNP, but also GDF15, are synthesized as prohormones and become active hormones. Insulin-like growth factor 1 (IGF1) signaling acts as an important modulator of cellular functions through its receptors: IGF1-R. Growth hormone (GH) secreted from the pituitary gland sends signals to the liver to stimulate the production of IGF1. In the blood, GDF15 levels act on the liver inducing an inhibition of the actions of GH (+: stimulation; −: inhibition; ↑: increase).

In the adult heart, mitotic division of cardiac myocytes is not easily detectable. The majority of mature cardiomyocytes are growth arrested in the G0 or G1 phase. The number of cardiomyocytes is stable in number, with a one-to-one ratio to the number of capillary microvessels providing oxygen and substrate delivery [13]. Cardiac fibroblasts are responsible for myocardial extracellular matrix homeostasis. They are central actors in normal cardiac physiology and play an essential role in development by depositing collagen and ECM factors. Under stress conditions, fibroblasts produce significantly more ECM proteins, which are responsible for the formation of fibrotic tissue (Figure 1). In mature hearts, fibroblasts are constantly modifying the microenvironment by degrading and removing ECM. The fibroblast is a plastic cell type, and the differentiation of cardiac fibroblasts into myofibroblasts has been demonstrated. In a pathological situation, for instance after myocardial infarction, fibroblasts are transdifferentiated into cardiac myofibroblasts. Myocardial insult activates fibroblasts, including fibro–adipogenic progenitors, leading to migration to the injury site and marked proliferation. Activation is associated with upregulation of activated fibroblast markers such as periostin, lysyl oxidase, and prolyl-4-hydroxylase. These cells play an essential role in the fibrotic healing response. Therefore, the transformation of cardiac fibroblasts into myofibroblasts is an important process in the adaptation of the myocardium, and this procedure is mediated by a range of chemical and physical stimuli [5,14].

During the development of the heart, the cardiac cells are exposed to mechanical forces. Cardiac myocytes and fibroblasts have been shown to respond to a variety of mechanical stimuli such as dynamic stresses, and a variety of molecular mediators have been identified. The transmission of forces between the interior and exterior of cardiomyocytes is facilitated by transmembrane proteins such as integrins. Mechanosensitive ion channels that regulate transmembrane fluxes of ions are localized in the sarcolemma and transverse tubule system in cardiac myocytes. Myocyte stretch can have direct effects on the nucleus via force transmission through the sarcolemma and cytoskeleton. The mechano-sensors arise inside the nucleus of cardiomyocytes and are able to modulate protein expression in the nucleus [15]. Evidence collected in recent years has confirmed that new cardiomyocytes can be generated in the adult mammalian heart. Maximal cardiac myocyte turnover during homeostasis in adults is limited to about 1% per year. In a context of cardiac injury or disease, this turnover marginally increases but cannot compensate for extensive myocyte loss. Replicating myocytes represent a short-lived cell pool that is continuously replenished by primitive cells and is constantly transitioning to the terminally differentiated phenotype [16].

In conclusion, progressive aging and pathological changes in cardiac homeostasis result in progressive alterations in the structure, metabolism, and function of the heart. The most important of these include a reduced number of myocardial cells and increased interstitial collagen fibers, which result in the development of heart failure (HF). Thus, to fully understand the biology and pathobiology of the heart, the influence of cellular crosstalk must be considered [17].

## 2. Cardiac Cellular Senescence and Tissue Regeneration

Advancing age is associated with the progressive alteration of blood vessels and myocardium, making them more vulnerable to stressors. The aged heart has increased mass, thickened ventricular walls, and increased cardiomyocyte cross-sectional area, despite a decrease in the number of cells [18]. Cellular senescence associated with aging is characterized by increased reactive oxygen species (ROS) production, and oxidative stress and DNA damage in response to a variety of physiological and pathological conditions [19,20].

**Figure 2:**
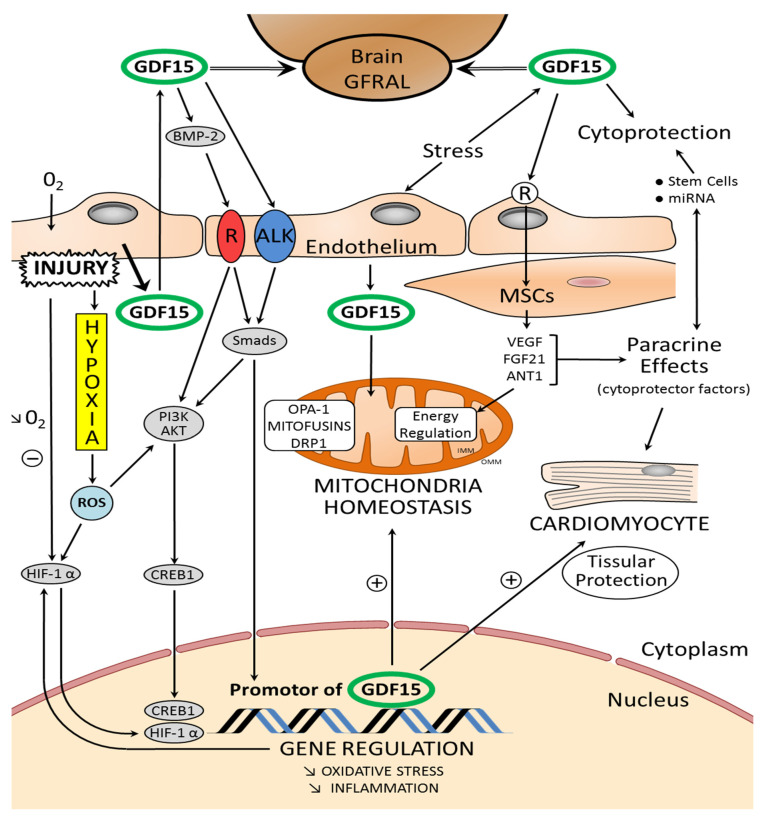
Schematic representation of the relationship between mitochondrial homeostasis and the properties of GDF15: GDF15 modulates mitochondrial functions by inducing mitochondria-related genes. OMM: outer mitochondrial membrane, IMM: inner mitochondrial membrane. In the figure, the main proteins orchestrating mitochondrial homeostasis are highlighted: DRP1, dynamin-related protein 1; Mfn 1–2, mitofusin 1 and 2; OPA-1: optic atrophy protein-1. Fibroblast growth factor 21 (FGF21) and adenine nucleotide translocator isoform 1 (ANT1) are regulators of mitochondrial metabolism. The paracrine mechanisms, mediated by factors released by the stem cells and implicating microRNAs (miRNA), play a role in cytoprotection of the myocardium. Exosomes, especially those secreted by different cardiac stem cells, confer cardioprotective effects. Stem cells and muscular smooth cells (MSC) secrete growth factors such as vascular endothelial growth factor (VEGF) and fibroblast growth factor-2 (FGF-2). Tissue damage and cellular stress induce the expression and secretion of GDF15 from various cells in the myocardium. Glial-derived neurotrophic factor (GDNF) family receptor α-like (GFRAL) is an endogenous receptor for GDF15; it is identified selectively in some areas of the brain. GDF15 interacts on endothelial cells with a receptor-pair consisting of activin receptor-like kinase (ALK). GDF15 regulates the signaling pathways that are essential for regulating proliferation and angiogenesis through activation of ALK receptors and phosphorylation of Smad. GDF15 activates PI3K/AKT. The AKT pathway is directly linked to C-AMP response element-binding protein (CREB1) activation. Subsequently, activated CREB1 acts as transcription factor in the nucleus by directly binding the promoter region of GDF15. After myocardial injury and hypoxia, hypoxia-inducible factors (HIFs) are molecular mediators of the response to hypoxia. HIF-1α is a transcription factor and GDF15 activates the HIF-1α signal (+: stimulation; −: inhibition; ↓: decrease).

Age is the most important risk factor for most diseases. DNA and mitochondria play a central role in bioenergetics and the adaptation of the cell to the environment. DNA lesions can persist over a long period of time and increase with age. In addition, several lines of evidence indicate that mitochondria have an influence on lifespan determination and aging. Mitochondrial dysfunction diminishes the functional performance of tissue and organs. Proper mitochondrial function and maintenance require the action of multiple mechanisms. Mitochondria are crucial organelles for the production of ATP, and their morphology and function are regulated by the dynamic processes of fusion and fission. The processes of fusion, fission, and mitophagy are tightly connected, determining mitochondrial morphology and dynamics [21]. Alterations in these processes impair mitochondrial homeostasis (Figure 2) and function with age and are linked to age-associated functional decline, such as the aging of the brain or heart [22]. Progressive alterations in the primary structure of the genomic DNA (nuclear and mitochondrial DNA: mtDNA) can compromise mitochondrial function in various ways. Mitochondria contain ~1200 proteins, only 13 of which are encoded by mtDNA. All of these proteins are components of the oxidative phosphorylation system and are essential for mitochondrial function [23]. Thus, cellular senescence might be an important consequence of age-related decline in mitochondrial homeostasis. Cellular senescence, which accompanies aging, is associated with an extensive loss of function at all levels of biological organization. Studies using model organisms have generated significant insights into the genetic factors and environmental conditions that, in the heart, influence the age-related decline of these functions and tissue regeneration [17,24] (Figure 1).

In the heart, tissue regeneration is characterized by complex cascades of endogenous factors with specific roles in cell proliferation and differentiation. The association of various growth factors is required to imitate the native environment and to stimulate the development of new functional tissue [25]. The myocardium is characterized by limited regenerative capacity. The neonatal mammalian heart can substantially regenerate after injury through cardiomyocyte proliferation until postnatal day 7 [26]. How the adult heart is able to regenerate cardiomyocytes or not is widely debated. Tissue homeostasis in skeletal muscle is fundamentally different from that in cardiac muscle. Skeletal muscle is able to self-repair efficiently. It has been reported that cardiomyocytes renew, with a gradual decrease from 1% annual turnover at the age of 25 to 0.45% at the age of 75 [27]. Recent studies revealed that mammalian cardiomyocytes retain some capacity for division and identified endogenous cardiac progenitor cells in the heart [28]. Mesenchymal stem cells (MSC) are adult stem cells with a capacity for self-renewal and multi-lineage differentiation. Initially described in the bone marrow, MSC are also present in other organs and tissues. MSC are emerging as an extremely promising therapeutic agent for tissue regeneration and repair. Studies in animal models of MI have demonstrated the ability of transplanted MSC to engraft and differentiate into cardiomyocytes and vascular cells. MSC secrete soluble factors that mediate beneficial paracrine effects and may greatly contribute to cardiac repair [29].

The recent discovery of resident cardiac stem cells (CSCs), and the demonstration of bone marrow (BM)-derived stem cells able to reside in the heart and transdifferentiate into cardiomyocytes, has raised the possibility that myocardial regeneration might be achieved using adult stem cells (ASCs). CSCs possess the properties required to achieve cardiac regeneration. The paracrine mechanisms mediated by factors released by ASCs play a major role in the repair process after stem cell mobilization. ASCs and MSCs secrete cytokines, chemokines, and growth factors such as vascular endothelial growth factors (VEGFs) and fibroblast growth factors (FGFs). These growth factors have well-established roles in angiogenesis. They are involved in cardiac regeneration, and hypoxia increases their production [30,31].

Cardiomyocyte proliferation is important for regeneration as well as postnatal heart growth. In mice, developmental cardiomyocyte proliferation continues for up to 7 days after birth, which coincides with the loss of regenerative capacity [26]. Studies in rodents and humans have demonstrated that, whereas embryonic, fetal, and early postnatal cardiomyocytes can divide, adult cardiomyocytes are predominately quiescent. This cell cycle arrest coincides with the postnatal switch from a hypoxic to an oxygen-rich environment [32]. Recent analyses have revealed that the cell renewal in the heart is primarily confined to the endothelial and mesenchymal cell populations and that there is a much more limited exchange of cardiomyocytes. ECs have the highest exchange rate, with the whole population being renewed every 6 years in adulthood [33]. As we reported, the adult mammalian heart has a limited capacity to regenerate after injury (Figure 1). After myocardial injury, the healing process is divided into three phases: inflammatory, proliferative, and maturation. Early inflammatory activation is a necessary event for the transition to the subsequent reparative and proliferative stages [34].

Finally, the findings from new “omics” approaches show that cardiomyocyte maturation is regulated at the level of the epigenome, coding and noncoding transcriptome, proteome, and metabolome. Concerning the hypotheses about how cardiomyocyte maturation is controlled, several new candidates associated with energetic metabolism have been confirmed as major regulators of maturation [24]. During these processes, the adaptations for metabolic changes appear to be essential. During embryonic development, the heart utilizes anaerobic glycolysis as a main source of energy, whereas adult cardiomyocytes utilize oxygen-dependent mitochondrial oxidative phosphorylation as the primary energy source accompanied with energetic advantage. In addition to the shift from cytoplasmic glycolysis to mitochondrial oxidative phosphorylation according to developmental age, postnatal cardiomyocytes exhibit a shift in energetic substrate utilization, from pyruvate to fatty acids [35]. Fatty acid utilization is energetically positive, and it also becomes abundant to neonatal mammals immediately after birth because of the high fat content in breast milk, which is as high as 30%. Mitochondrial-derived ROS induce DNA damage and activate DNA damage response (DDR) in the early postnatal heart, resulting in cell cycle arrest [32]. In rodents, the developmental timing of regenerative arrest coincides with the postnatal window when most cardiomyocytes withdraw from the cell cycle and become terminally differentiated.

Myocardial metabolism changes throughout development from the fetal stage to adulthood. At the fetal stage, the low level of fatty acids and high level of lactate in the blood activate anaerobic glycolysis as the major source of ATP production in the heart. During development, the increase in dietary lipid levels and oxygen concentrations in circulating blood are important in mediating metabolic reprogramming [36]. Genetic studies in mice have identified cardiomyocyte proliferation as the primary source of regenerated cardiomyocytes. Neonatal mice fed fatty acid-deficient milk showed prolongation of the postnatal cardiomyocyte proliferative window. Inhibition of fatty acid utilization in cardiomyocytes promotes proliferation and may be a viable target for cardiac regenerative therapies [37].

## 3. Cardiac Remodeling and Progressive Heart Dysfunction

Throughout life, persistent stress leads to ultrastructural remodeling in which cardiomyocyte death exceeds cardiomyocyte renewal, resulting in progressive heart dysfunction. Recent studies have revealed that mammalian cardiomyocytes retain some capacity for division and identified endogenous cardiac progenitor cells in the heart. Cardiac progenitor cells (CPCs) show great potential as a cell supply for restoring cardiac function in patients affected by heart disease. CPCs are proliferative and able to generate cells from all of the cardiac lineages [38]. The epicardium, a mesothelial cell layer, is a source of progenitor cells that are essential for heart development, repair, and regeneration during HF. Signaling molecules released by the epicardium influence the underlying myocardium. During embryogenesis, the epicardium generates progenitor cells that differentiate into vascular SMCs, cardiac fibroblasts, and ECs [39]. The innate capacity of cardiomyocytes to self-replace is insufficient to compensate for the large-scale tissue damage associated with MI. Among the cardiovascular diseases associated with MI, HF is a typical age-related disease, and the number of patients with HF increases exponentially with aging. A major cause of heart diseases such as HF is the massive loss and/or dysfunction of cardiomyocytes, which may be caused by clinical interventions such as cancer treatments [40]. In the physiopathology of cardiac diseases, many studies have confirmed the role of inflammatory cells. ECM contributes to the regulation of inflammation and repair, mediating cardiac remodeling. Both the positive and negative roles of inflammation have been demonstrated in tissue repair and regeneration [41].

Multiple levels of regulation have been described in the cardiac cell cycle, including classic regulators such as cyclins, but also by miRNA. Given the importance of miRNAs in cardiac function and their role in various cardiovascular diseases, numerous studies have sought to determine the therapeutic potential of miRNAs. Therapeutic approaches using miRNAs are separated into several categories: miRNA overexpression (agomiRNAs), miRNA inhibition (blockade) to reduce miRNA expression, and miRNA replacement to restore the expression of miRNAs [42]. Recently, a link has been established between microRNA and mitochondrion in HF. It was demonstrated that sixty-nine microRNAs were upregulated and two were downregulated in early HF, while sixteen microRNAs were upregulated and six were downregulated in late HF. Differential microRNA expression profile analysis for mitochondria microRNAs has been demonstrated during development of HF [43]. However, the potential function of these miRNAs in HF is not clear. A number of studies have examined circulating levels of microRNAs, including cardiomyocyte-enriched microRNAs, in the plasma or serum of patients with acute HF [44]. Moreover, it was recently suggested that the miRNA-132/212 family is a novel regulator of cardiovascular function, including the regulation of vascular adaptation, cardiac hypertrophy, and HF [45]. In the myocardium, coronary injury triggers phenotype changes in vascular SMC. The modified phenotype is characterized by inappropriate contractility, increased proliferation, and migration resulting in restenosis. The miRNA-212/132 cluster is a modulator of the VSMCs phenotype, which prevents proliferation and migration as well as the release from VSMCs of pro-inflammatory biomarkers. In rats, miRNA microarrays showed an upregulation of miRNA-132 in the carotid artery after catheter injury [46].

## 4. Basic Biology of GDF15

### 4.1. Synthesis, Structure, Secretion, and Distribution of GDF15

Growth and differentiation factor 15 (GDF15) (also known as macrophage inhibitory cytokine-1 (MIC-1), placental transformation growth factor (PTGF-b), prostate derived factor (PDF), placental bone morphogenetic protein (PLAB), prostate derived factor, or non-steroidal anti-inflammatory drug activated gene-1 (NAG-1)), belongs to the transforming growth factor-β (TGF-β) superfamily of proteins. The TGF-secreted factor superfamily involves more than 30 members. The TGF-β superfamily comprises numerous ligands, including TGF-βs, bone morphogenetic proteins (BMP), activins, and GDFs. GDFs belong to the activin/myostatin (MSTN) subclass. The elements of this subfamily have been designated GDF1 through GDF15 [47]. TGF-β family proteins bind to distinct type I and type II serine/threonine kinase receptors. Signaling induced by the TGF family ligands are implicated in various processes during vertebrate development, organ repair, and tissue homeostasis [48,49,50].

The human GDF15 locus was plotted by fluorescence in situ hybridization (FISH) to chromosome 19p12.1-13.1; the gene contains a single 1820 bp intron [51]. Specific variants near the GDF15 gene on chromosome 19p13.11 were intensely associated with GDF15 concentrations [52]. In a recent genome-wide association study (GWAS) using a sample of ∼5400 community-based Caucasian participants, the genetic variants associated with GDF15 blood concentrations were evaluated. Gene-based analysis confirmed the chromosome 19 locus association and, in addition, a putative locus on chromosome 1. A locus on chromosome 19 was associated with GDF15 blood concentration with genome-wide significance, with evidence for a new locus: chromosome 1 [53].

A number of polymorphisms have been identified in the GDF15 gene; the aim of a recent clinical study was to evaluate the relationship between GDF15 gene polymorphisms, GDF15 levels in the blood, and the development of specific disorders. The distribution of the rs1804826G/T polymorphism was significantly different between two groups of patients, and the data suggest that the GDF15 gene may play a role in the development of ischemic stroke. It is hypothesized that the rs1804826G/T polymorphism contributes to variant molecule consequences, and induces an impact on the “dynamic” of mRNA, thereby modifying the production of GDF15.

ProGDF15 is a precursor protein of GDF15, which undergoes disulfide-linked dimerization (a 25-kDa disulfide-linked dimer), as TGF-β does. The unprocessed translated form of GDF15 (pre-pro-GDF15) is 308 amino-acids (aa) long, including the signal sequence (29 aa), the propeptide (167 aa), and a mature protein (112 aa) that contains a cysteine knot typical of the TGF-β family. GDF15 is produced as a ≈40 kDa propeptide form. The mature protein is secreted as a homodimer linked by disulfide bonds and is released from the propeptide following intracellular cleavage. GDF15 is soluble and may be evaluated in the blood. The mature peptide is an endogenous compound with a broad normal circulating range of ~0.15–1.15 ng/mL in humans without disorders. GDF15 concentrations increase with age. Previous studies have reported that GDF15 concentrations are accompanied by cystatin C and C-reactive protein concentrations [54,55].

### 4.2. Distribution of GDF15

GDF15 is widely expressed at different levels in various human tissues, including the placenta, kidney, lung, pancreas, heart, skeletal muscle, liver, and brain. Various reports have shown an association between GDF15 and aging and longevity. GDF15 has been investigated for its contribution to aging and age-related cognitive decline [56]. One recent study assessed whether baseline levels of GDF15 were associated with total mortality in community living older adults over eight years of follow-up. In this context, GDF15 was significantly correlated with gait speed, hand grip strength, and walking duration [57]. Over the past years, a great number of genetic studies in animal models have sought to clarify the molecular mechanisms of the role of endogenous factors, in particular GDF15, in aging. In this field, it has been demonstrated that female transgenic mice overexpressing GDF15 in various tissues, such as skin, colon, kidney, brain, and adipose tissues, have prolonged lifespans [58].

In humans, GDF15 is widely expressed in skeletal muscle and circulating GDF15 increases during exercise and recovery from exercise. It is conceivable that GDF15 is secreted from muscle in response to cellular stress and injury in order to act nearby [59]. Concerning the heart, GDF15 is not normally expressed in the adult myocardium, although it is significantly induced after “injury” or in heart failure. A number of fundamental research studies have identified proteins produced by the heart, referred to as cardiokines, which may function analogously to adipokines. Cardiokines are proteins secreted by the heart, and they play a physiological role in maintaining heart homeostasis or reacting to myocardial damage. Cardiokines may act as biomarkers to evaluate cardiac function, and therefore contribute to clinical diagnosis. Through paracrine/autocrine pathways, they affect the response of cardiomyocytes and cardiac fibroblasts to pathological abnormalities caused by heart damage or other inflammatory processes. In cardiac fibroblasts, TGF-β is involved in many aspects of fibrosis, including inflammation, gene expression, and ECM synthesis. TGF-β receptors signal via Smad proteins, which translocate to the nucleus and regulate gene transcription. Cardiac fibrosis and the accumulation of ECM are present in almost all forms of cardiac disease. In the diseased myocardium, the balance between profibrotic and antifibrotic processes is influenced by hemodynamic, humoral, and metabolic factors. Myocardial fibrosis is histologically defined as the diffuse deposition of type III collagen fibers within the myocardial interstitium. The collagen is managed and/or destroyed by matrix metalloproteinases (MMPs). Recently, plasma levels of GDF15, MMPs, tissue inhibitors of MMP 1 (TIMP1), and soluble suppression of tumorigenicity-2 protein (sST2) were determined in patients with idiopathic dilated cardiomyopathy. Significant associations were found between GDF15 and MMPs, sST2, HT parameters, and NYHA class [60]. Heart diseases such as HF are complex syndromes where interactions between all of the myocardial cells subjected to inflammation and remodeling contribute to the pathophysiology and progression of the disease.

In the myocardium, cardiomyocytes and fibroblasts are the main sources of inflammatory signals, contributing to the pro-inflammatory environment by the production of various cardiokines, including GDF15 [61]. During myocardial injury, GDF15 is expressed in all myocardial cells: cardiomyocytes, adipocytes, macrophages, ECs, and vascular SMCs [62,63].

In certain conditions, GDF15 causes endothelial dysfunction (Figure 2) by impairing vascular contraction and relaxation, which could have deleterious consequences on cardiac function by inducing microvascular disease, which is associated with deteriorations in heart function [64]. Various studies show associations between GDF15 and cardiac fibrosis during HF and MI. Some demonstrated a correlation between circulating GDF15 and the severity of fibrosis, indicating that GDF15 is involved in the process of fibrosis. Within the heart, this fibrosis is thought to be partially mediated by TGF-β1, a potent stimulator of collagen-producing cardiac fibroblasts. However, the origin of this increased GDF15 production is not clearly identified. Cardiac mRNA and protein expression of GDF15 are very low, suggesting that the heart is not an important producer of GDF15 in these patients [65]. Further research is needed to identify the specific effects of GDF15 on cardiac fibrosis.

One recent study has investigated GDF15 expression induced by radiation. The deleterious effects of radiation are well known. An adverse, late-onset side effect of thoracic radiotherapy is the development of radiation-induced heart disease (RIHD). Two major components are thought to be responsible for the development of RIHD in humans. The first is progressive atherosclerosis of the coronary arteries due to endothelial damage, and the second is injury and inflammation of the myocardium [66]. These mechanisms could contribute to cardiomyocyte hypertrophy and interstitial fibrosis in the early stages of RIHD, thus inducing HF. In recent work focusing on the effects of radiation, the objective was to elucidate the underlying molecular mechanisms and to identify genes implicated in cardiac calcium homeostasis (PDE3B), oxidative stress response (FDXR and SPATA18), and the etiology of cardiomyopathy (SGCD, BBC3, and GDF15). The analysis of upregulated genes suggested that GDF15 was a candidate key player in the response of cells to radiation [67]. It is not clear if GDF15 can counteract radiation-induced cardiotoxicity in these conditions by inhibiting macrophage-dependent inflammatory and fibrotic pathways or if the induction of GDF15 is a marker of the injury.

### 4.3. GDF15 and Cellular Senescence

GDF15 is thought to be associated with aging, cognitive decline, and neurodegenerative diseases. Clinical studies have shown the association between GDF15 and aging. GDF15 concentrations are predictors of all-cause mortality [68]. Inflammatory markers are elevated in the elderly and are associated with senescence. The mechanism of the cellular senescence is a complex stress response associated with a modification of expression of senescence regulators including GDF15 [69,70,71]. Cellular senescence was reversed by the downregulation of GDF15; GDF15 overexpression induced growth arrest and cellular senescence [71]. One of the main functions of TGF-β is its cytostatic effect that is dependent on the cell type. Among the TGF-β family of proteins, the pharmacology of GDF15 and GDF11 is raising exciting prospects for the development of anti-aging therapeutics and protection against senescence [50,72]. Another factor that mediates rejuvenating actions has been identified in mouse blood; it is a chemokine (C–C motif) chemokine 11: CCL11. In contrast to levels of GDF11, levels of CCL11 increase with age [73].

Another important subject relative to the biological activity of GDF15 is tumor genesis. Several studies report higher expression of GDF15 mRNA and protein in cancer biopsies. GDF15 is a secreted protein induced by the tumor suppressor protein, which is implicated as a growth inhibitory molecule in tumor cells. This effect is associated with activating transcription factor 3 (ATF3), a pro-survival protein that is negatively regulated by p53 protein expression [74]. The tumor suppressor protein p53 is a redox active transcription factor that can interact with ROS indirectly through signaling networks or directly through the redox-sensitive thiol groups (–SH) of cysteines (Cys) located in the DNA-binding domain of p53. Recently, it has been demonstrated that the cardioprotection induced by angiotensin II receptor antagonist was attributable, at least in part, to a reduction in apoptosis induced by downregulating p53 and the decrease in inflammation induced by an upregulation of GDF15. The properties of P53 and GDF15 work in coordination in the myocardium [75].

The expression of GDF15 appears to be regulated in various tissues and phases of development by AKT1 and ERK1/2 through the various cellular mechanisms of the PI3K/AKT and MAPK/ERK signaling pathways. The AKT pathway is directly linked to C-AMP response element-binding protein (CREB1) activation. Subsequently, activated CREB1 acts as a transcription factor by directly binding the promoter region of GDF15. We reported previously that NRG-1/ErbB signaling was involved in a multitude of cardiac processes with protective effects following injury. GDF15 and NRG-1/ErbB are determinants of heart development and angiogenesis. In addition, GDF15 and the ErbB2 receptor are implicated in cancer proliferation. GDF15 promotes the proliferation of cervical cancer cells by phosphorylating AKT1 and ERK1/2 through the ErbB2 receptor. ErbB2, which is implicated in cell proliferation, migration, and differentiation, is transactivated by GDF15 in human cancer cells [76]. Some cellular environmental conditions are able to promote tumor growth via the increased release of GDF15. The interactions between a developing tumor and its microenvironment are known to implicate a complex “crosstalk” among the factors produced by the population of cells. GDF15 can modify the tumorigenesis both positively and negatively [77].

## 5. GDF15 Signaling—Molecular Mechanisms Underlying GDF15 Activity

### 5.1. GDF15: Interaction with Smad Signaling and miRNA

In the adult heart, TGF-β-mediated signaling, through the receptor-activated Smads, regulates some cellular functions. The main attribute of the TGF-β signaling pathway is context-dependence. Depending on the concentration and type of ligand, target tissue, and developmental stage, TGF-β family members transmit distinct signals. Once located in the nucleus, Smad complexes can directly bind DNA and modulate transcription (Figure 2). Smad complexes bind with low affinity to a DNA sequence known as the “Smad binding element” (SBE). The intracellular mechanism of the TGF-β family is divided into Smad dependent and Smad independent pathways, and is determined by the type 1 receptors (ALK1 to 7). GDF-15 activates type 1 receptors and phosphorylates Smad2/3 and Smad1/5/8, which translocate to the nucleus in the form of heteromeric complexes with Smad4. The Smad dependent pathway can inhibit apoptosis and protect against hypertrophy and fibrosis. GDF-15 was found to inhibit myocardial hypertrophy through a Smad2/3 pathway [78].

Smad complexes control the transcription of miRNA genes by binding to SBEs in their promoter. In addition, Smads can indirectly modulate miRNA levels through activation of transcription factors that regulate miRNA promoter activity. The regulation of genes by miRNAs is an integral component of the control of gene expression exerted by the TGF-b family of ligands [79]. MiRNAs control various processes such as cellular growth, proliferation, differentiation, regulation of the cell cycle, aging, inflammation, and immune responses. Many studies have shown that miRNA dysfunction is closely related to the occurrence and development of diseases.

Recent results established a pathway between the functions of some miRNAs and the cellular properties of GDF15 in relation to certain diseases. We previously reported that miRNAs have a key role in cardiac function and described their role in various cardiovascular diseases. In humans and old-world primates, a 73-base-pair transcript encoding miR-3189 is found in the GDF15 intron. Existing evidence suggests that this intronic miRNA is co-transcribed with the GDF15 gene. It has also been suggested that miR-3189 induces GDF15, but the mechanism underlying this effect is unknown [80]. Recent high-throughput RNA sequencing studies have provided evidence supporting the existence of the putative miR-3189-3p embedded in the intron of the p53-inducible TGF-β superfamily member GDF15 on chromosome 19p13.11. Experimental studies have shown that the knockdown of GDF15 inhibits growth, whereas antagonizing miR-3189-3p causes an increase in proliferation. miR-3189-3p is thought to be integral to the cellular response to genotoxic stress, and miR-3189-3p may also be involved in the response to hypoxia or inflammatory stress, which have also been shown to upregulate GDF15 [80].

As we report later in this review, novel biomarkers, including miRNA and GDF15, are clinically available but not yet routinely used. However, they provide additional information in terms of diagnosis, severity, and prognosis of cardiovascular disease [81].

### 5.2. Molecular Mechanisms of GDF15 and Oxygen Metabolism

Oxygen availability is a determinant of heart development and angiogenesis, implicating various endogenous factors such as hypoxia-inducible factor (HIF) (Figure 2). HIF is a major transcription factor in various tissues. It consist of three α-subunits (HIF1α, HIF2α, and HIF3α) and one β-subunit (HIF1β), which serves as a heterodimerization partner for the HIFα subunits. They modulate the expression of many oxygen-sensitive genes such as VEGFs, and also the TGF-β superfamily, including GDF15. These pro-angiogenic growth factors are upregulated, along with their receptors, in response to cardiac conditions such as ischemia, in a process mediated by HIFα.

The hypoxic environment of the embryo supports the function of the HIF-1α transcription factor and the expression of genes involved in angiogenesis and fetal heart remodeling. Its expression is high during gestation and decreases postnatally. GDF15 activates HIF-1α signaling via stabilizing p53/mouse double minute 2 homolog (MDM2) complex [82] and activation of Akt, MAPK, and nuclear factor κB (NF-κB) signaling [83]. After birth, exposure to high oxygen levels limits HIF-1α function and allows three key regulators of mitochondrial dynamics to be expressed: optic atrophy type-1 GTPase (OPA-1), mitofusins (Mfns 1 and 2), and dynamin-like GTPase dynamin-related protein 1 (DRP1). These regulators initiate the postnatal maturation of mitochondria [84]. The OPA-1 mutation induces late-onset cardiac dysfunction, with no changes in the early stages of life, whereas 12-month-old Opa1+/− mice show decreased fractional shortening, cardiac output, and myocyte contraction; by contrast, OPA-1-overexpressing mice (Opa1tg) show physiological heart hypertrophy at 9 months [85]. Mfns were named for their ability to promote mitochondrial outer membrane fusion; they also act as mitochondria–mitochondria ropes. The deletion of either Mfn1and Mfn2 produces different effects in cardiomyocytes. Ablation of Mfn2 in mice causes mitochondrial enlargement and ROS production, leading to further cardiac hypertrophy. The ablation of Mfn2 could be protective in cardiomyocytes [86]. Mitochondrial fission is largely dictated by DRP1, which is mainly located in the cytosol under basal conditions and is recruited to the outer membrane of mitochondria. Increased mitochondrial fission has been documented in models of ischemia/reperfusion, and pharmacological inhibition of DRP1 reduces infarct size in mice subjected to coronary artery occlusion and reperfusion. Data suggest that mitochondrial fission plays a role in the development of cardiac diseases, and the potential of preserving mitochondrial fusion as an approach to cardioprotection has been demonstrated [87]. In conclusion, cardiac dysfunction is accompanied by a decline in mitochondrial function, dysregulation of mitochondrial fusion/fission, and increased production of ROS. Regulators of mitochondrial dynamics are important for mitochondria remodeling. Cardiac mitochondria undergo major adaptations throughout the life cycle [88].

Mitochondrial stress and mitochondrial adaptation elicit the production of response molecules designated as mitokines. A mitokine is a soluble molecule (protein, peptide, or other) produced and secreted in response to a mitochondrial stress response and able to elicit an adaptive/compensatory response. Since the description of mitokines was established, a number of molecules, including GDF15, have been identified as such. GDF15 is induced in the cells by hypoxia, and this effect is related in various tissues. In vitro, both BEAS-2B (human bronchial epithelial cells) and human pulmonary vascular endothelial cells (HPMEC) showed a significant increase in GDF15 expression upon exposure to an oxygen deficit. After GDF15 knockdown, there was a significant decrease in cell viability and an increase in oxidative stress compared to control cells transfected with siRNA with a scrambled sequence [89].

### 5.3. GDF15: A Regulator of Mitochondrial Functions

It appears that GDF15 may control mitochondrial functions through the induction of mitochondria-related genes. In mice in which the heart–muscle–brain adenine nucleotide translocator isoform 1 (ANT1) was inactivated, the muscle mitochondria produced excess ROS. Muscle transcriptome analysis demonstrated an induction of mitochondrial biogenesis and increased the expression of genes encoding FGF21 and GDF15 [90]. FGF21 is a regulator of metabolism and performs an important role in glucose and lipid metabolism and the maintenance of energy balance. FGF21 is a key metabolic mediator to improve the altered mitochondrial function. It reduces inflammation and apoptosis in several organs.

Mitochondrial myopathy was promoted in mice in which the ANT1 gene was deleted in relation to the hyperproliferation of skeletal muscle mitochondria [91]. While there is currently no reliable biomarker for the screening or diagnosis of mitochondrial diseases (MDs), GDF15 may be a useful novel biomarker for the specific screening of MDs because its measurement involves a less-invasive approach than muscle biopsy. GDF15 concentrations were evaluated from patients diagnosed with MDs, controls with other diseases, and healthy controls, and evaluated for their ability to identify MD. Serum levels of GDF15 were 6-fold higher in MD patients compared to healthy controls. In this context, muscle mitochondrial stress is correlated with the induction of GDF15 [92]. Intriguingly, plasma GDF15 was recently reported to be increased in MD patients, who experience slower-than-expected growth, organ failure, and muscular dystrophy. Among the various mitochondrial disorder subtypes, GDF15 concentrations were 10-fold higher than controls [92]. The mechanism that leads to elevated levels of GDF15 in MDs is not clear; however, in this context, GDF15 is a useful biomarker for muscle-related disease. It has been suggested that the mitochondrial dysfunction induced by alcohol and carbon tetrachloride (CCl4) promotes GDF15 production in hepatocytes and cardiomyocytes. It has been demonstrated that recombinant GDF15 (rGDF15) decreased the expression of pro-inflammatory cytokines and prevented the activation of T cells in the livers of mice with CCl4-induced liver fibrosis [93]. To conclude, an interesting new hypothesis is that GDF15 could be used as a marker for the diagnosis of mitochondrial diseases.

It is now speculated that circulating GDF15 levels might reflect general mitochondrial dysfunction in aging and age-related disorders. Mitochondrial stress elicits, as reported previously, the production of stress response molecules such as mitokines, including GDF15 and a number of mitochondrial peptides encoded by mitochondrial DNA [94]. The mitochondria produce mitochondrial-derived peptides (MDPs), specific peptides that mediate transcriptional stress responses by translocating into the nucleus and interacting with DNA. MDPs are regulators of metabolism. This class of peptides comprises: humanin (HN), mitochondrial open reading frame of the 12S rRNA-c (MOTS-c), and small HN-like peptides (SHLPs) [95]. These proteins are produced in response to mitochondrial stress. They have antiapoptotic and cytoprotective roles in several age-related diseases. Recent data indicate that the circulating levels of GDF15 and HN are associated with worse health and mortality in old age, the predominant association being observed for GDF15 [96].

Given the associations of GDF15 with a variety of biological processes including metabolism, there are a number of open questions raised by recent studies in relation to mitochondrial disorders. Mitochondrial diseases primarily affect organs and tissues with high energy requirements and they tend to have prominent neurological and cardiovascular manifestations. The interpretation of the different results in this field is not straightforward.

In a recent study, plasma GDF15 concentrations were assessed in patients with genetically confirmed primary mitochondrial disease and patients with non-mitochondrial inherited diseases. Elevated GDF15 is not specific to mitochondrial diseases neither is it a diagnostic biomarker; GDF15 is therefore not acceptable as a diagnostic test for mitochondrial diseases such as spinal muscular atrophy and Duchesne muscular dystrophy [97]. The results of a more recent study [98] clearly demonstrated a significant increase in GDF15 in the serum of individuals with mitochondrial disease, which significantly differentiated the mitochondrial DNA genetic defects group from the nuclear DNA genetic defects group. Cell free circulating-mtDNA (ccf-mtDNA), a new biomarker for mitochondrial diseases [99], and GDF15 levels were significantly increased in patients with mitochondrial encephalomyopathy lactic acidosis stroke-like episodes syndrome (MELAS) and myoclonic epilepsy ragged red fibers (MERRF) syndrome. Additional studies are needed to clarify the origin and the role of GDF15 in mitochondrial diseases in relation to the pathogenic mechanisms and disease progression. It is important to clarify the specific function of GDF15 in mitochondrial diseases. A prospective cohort study has confirmed that GDF15 induction is highly restricted in patients with muscle disorders caused by defects in mtDNA expression [100].

GDF15 may be adopted as a novel diagnostic biomarker of mitochondrial diseases. In previous studies in humans with MDs, GDF15 mRNA was dramatically increased in thymidine kinase 2 (TK2)-deficient muscle and induced by the transcription factor p53 in response to various types of stress, such as DNA damage, oxidative stress, and metabolic stress. A TK2-deficient mouse strain exhibited growth retardation and several organ diseases, including in the skeletal muscle and heart. It is therefore possible that serum GDF15 originated from the muscle and heart, which are associated with the development of mitochondrial dysfunction. GDF15 may be a sensitive biomarker not only for primary MDs but also for dysmetabolic myopathies and cardiomyopathies [101].

### 5.4. GFRAL: An Orphan Member of the GDNF Receptor Family Identified as the Receptor for GDF15

Recently, four research groups from different pharmaceutical companies simultaneously identified GDNF family receptor α –like (GFRAL) as the receptor for GDF15 signaling though the RET coreceptor. RET is the abbreviation for “rearranged during transfection”. Alternatively, several laboratories have shown that both GDNF and GFRAL signal independently of RET [102,103,104,105].

GFLs, the GDNF family of ligands, act as biologically active homodimers that signal canonically through the RET transmembrane receptor. The stoichiometry of the GFL–GFRα– RET binding interaction is thought to be one ligand homodimer to two GFRα molecules to two RET receptors, forming a heterohexameric complex [106,107].

Among the areas in the CNS, GFRAL mRNAs were present more in some portions in the brain: the substantia nigra, the hippocampus, and the area postrema [108]. GDF15 activates GFRAL-expressing neurons (Figure 2) localized exclusively in the area postrema and nucleus tractus solitarius of the mouse brainstem [103]. GDF15–GFRAL signaling controls body weight. In normal conditions, the regulation of energy balance by the brain requires specific pathways concerning the influx of energy. Recent research has identified the molecular signaling pathways through which the brain and the gastrointestinal system communicate to govern energy homeostasis [109]. rGDF15 induces weight loss in mice fed a high-fat diet and in nonhuman primates with spontaneous obesity. Mice with a germline Gfral gene deletion (Gfral−/−) lost the anorexic and metabolic effects caused by rGDF15. Moreover, diet-induced obesity and insulin resistance were intensified in these mice, signifying that this receptor has a homeostatic role in metabolism [104].

## 6. GDF15 as a Metabolic Regulator in Relation to Inflammation

Because it functions as a metabolic regulator, GDF15 plays a pivotal role in the development and progression of diseases. GDF15 is expressed and secreted in response to oxidative stress and inflammation.

In patients with conditions such as diabetes, the hyperglycemia-driven excess generation of ROS induces oxidative stress in a variety of tissues. Oxidative stress is closely associated with chronic inflammation and has a key role in the pathogenesis of vascular complications [110]. Various studies have investigated the importance of both HO-1 and biliverdin (BVR) in the pathophysiology and therapy of diabetes-associated inflammation. However, the complex association between increased oxidative stress and increased inflammation makes it difficult to establish the temporal sequence of the relationship. HO-1 expression in ECs leads to decreased expression of vascular cell adhesion molecule-1 (VCAM-1) and the production of pro-inflammatory cytokines (monocyte chemoattractant protein-1; MCP-1). Upon activation of ECs, blood macrophages are recruited to the injury site, attach to the endothelium, and transmigrate to the subendothelial space, where they differentiate into macrophages. HO-1 is upregulated in macrophages during the development of inflammation [111].

Concerning the mitochondrial metabolism, a positive relationship between GDF15 and type 2 diabetes mellitus (T2DM) has been shown. The main hallmarks of the pathologic metabolic milieu of diabetes are hyperglycemia, insulin resistance, and pathologic lipid metabolism. The biochemical, cellular, and pathophysiological changes lead to endothelial dysfunction including a low-grade prothrombotic and major inflammatory state, impairing micro- and macro-circulation [112,113].

In these diabetic patients, the expression and secretion of GDF15 in myocytes were associated with altered mitochondrial oxidative phosphorylation function [114] suggesting that GDF15 is implicated in the regulation of energy homeostasis. It has been hypothesized that GDF15 is produced by ECs in response to a vascular stress, possibly to attenuate endothelial loss of function. GDF15, released from endothelium and adipose tissue, may act as a metabolic regulator. GDF15 also acts as an adipokine, similar to adiponectin and leptin [115]. Accumulating evidence shows that GDF15 could be associated with the development and prognosis of T2DM, but there is no evidence that elevated GDF15 has a predictive role in T2DM. Observational epidemiological studies noted a strong association between metformin use and the prevention of T2DM onset in patients at high risk. Metformin elevates circulating levels of GDF15, which is necessary to obtain its beneficial effects on energy balance and body weight (GDF15 mediates the effects of metformin on body weight and energy balance) [116]. Thus, GDF15 appears to protect the endothelium. GDF15 directly modulates vascular contraction and relaxation responses in an endothelium-dependent fashion that involves the nitric oxide (NO) pathway. This may be explained by the interference of GDF15 in the endothelial NO synthase (eNOS) associated with caveolin (CAV)-1 eNOS-CAV-1 interaction [64]. It has been reported that GDF15 stimulates eNOS and increases NO production [117].

Studies focused on the inflammatory process have identified complex interactions between enzymatic activity, erythropoiesis, iron metabolism, hepcidin, and GDF15 [118,119]. Hepcidin is involved in iron homeostasis and is stimulated by inflammation. Erythropoiesis inhibits hepcidin synthesis, thereby promoting iron absorption and increasing the levels of circulating iron available. Recently, it has been demonstrated that hepcidin expression is suppressed by GDF15, thereby increasing iron availability for hemoglobin synthesis [120]. GDF15 can be significantly upregulated by anemia and hypoxia [121]. Finally, GDF15, by affecting iron status, might be involved in the pathogenesis of anemia in patients with cardiovascular diseases.

A very important study was recently carried out to better understand the metabolic changes that occur in response to inflammation and infection. GDF15 is induced upon bacterial and viral inflammation and promotes metabolic adaptation to systemic inflammation [122]. Inflammation induced GDF15, and GDF15 are necessary for surviving both bacterial and viral infections, as well as sepsis. The protective effects of GDF15 were largely independent of pathogen control or the magnitude of the inflammatory response, suggesting a role in disease tolerance. The authors of this study suggest that GDF15 is a key component of the stereotypical inflammatory response and that it plays a role in metabolic adaptation to acute inflammatory stress. GDF15 is involved in coordinating at least one aspect of the physiological response to inflammation through the control of triglyceride availability. GDF15 stimulates hepatic triglyceride export via beta-adrenergic signaling. Finally, these data demonstrate that GDF15, which is induced after acute inflammatory insult, initiates hepatic triglyceride metabolism by targeting sympathetic outflow to the liver. A previous work reported that beta-adrenergic receptors are important in hepatic triglyceride metabolism during inflammation [123].

## 7. Role of GDF15 in Adapting the Body to Metabolic Conditions: Effect on the Homeostasis of the Heart

Cardiac development and adaptation to the environment are governed by a complex network of transcription factors that regulate cell fate in a spatiotemporal manner. In the postnatal heart, these factors play significant roles in cardiac function, regulating adaptive stress response, including cardiomyocyte hypertrophy and survival, as well as endothelial homeostasis and angiogenesis. In this area, GDF15 GATA4 has several wide-ranging roles in cardiac homeostasis, taking into account it is a major regulator of hypertrophic, angiogenic, and survival pathways.

GDF15 synthesis and secretion by cardiomyocytes are involved in the development of heart diseases. GDF15 was recognized as a cardiac hormone implicated in the regulation of body growth in association with the actions of the growth hormone (GH). GH, which is secreted from the pituitary gland, sends signals to the liver to stimulate the production of IGF1 and IGF-binding protein (IGFBP) via the JAK2-STAT5 pathway [124]. It has been demonstrated that GDF15 is required for the inhibition of liver GH signaling in failure to thrive associated with pediatric heart disease, and that children with concomitant heart disease and failure to thrive have elevated plasma GDF15. This suggests a new endocrine mechanism in which an endogenous pathway coordinates cardiac function and body growth. In these conditions, the heart synthesizes and secretes “hormones” to inhibit body growth, adapting the body to various mechanical adjustments. Among these hormones, ANP, BNP, and GDF15 are synthesized as prohormones and processed to become active hormones [62].

In relation to GDF15, GH, and IGF1 signaling are among the mechanisms that most strongly regulate postnatal growth. GH achieves its effects by influencing gene expression profiles, and IGF1 is a key transcriptional target of GH signaling in the liver. Circulating GDF15 affects the liver, inhibiting the actions of GH. These discoveries are important relative to clinical conditions such as pediatric heart disease. However, it is still not clear how GDF15 blocks GH signaling [125].

GDF15 is upregulated by various cardiovascular events associated with oxidative stress, as reported in coronary heart diseases (CHD), HF, and atherosclerosis [62]. In a pressure-overload murine model, GDF15 was also induced during cardiac hypertrophy, and its cardiac-specific overexpression protected the heart from hypertrophic responses [78]. Chronic HF patients had increased GDF15 concentrations that were closely related to disease severity. In mice, increased cardiac GDF15 concentration was reported in HF [78].

Several studies have investigated the potential value of GDF15 as a biomarker in the general population. The DAN-MONICA (Danish-Multinational MONitoring of trends and determinants in Cardiovascular disease) cohort illustrated the predictive role of repeated blood GDF15 measurement for death due to CHD and the occurrence of HF. In this clinical study, GDF15 was found to provide relevant prognostic information, suggesting that it is a promising biomarker for prediction of HF and death due to CHD in the general population [126]. A systematic review investigated the utility of GDF15 as a biomarker in HF. The analysis of 21 original studies (n = 20,920 study participants) showed that, in addition to the usual cardiovascular risk factors and biomarkers, GDF15 has added value in predicting all-cause mortality in HF patients [127].

The role of GDF15 is also essential for the association between diastolic function and HF. GDF15 may make it possible to distinguish normal diastolic function from asymptomatic diastolic dysfunction, and increasing GDF15 concentrations are associated with increasingly severe diastolic dysfunction [128,129]. Increasing plasma GDF15 concentrations have been reported in the various stages of HF. When compared with control subjects, preclinical HF was characterized by significantly increased plasma GDF15 concentrations and left ventricular mass index. GDF15 and N-terminal pro-brain natriuretic peptide (NT-proBNP) are reported to have equal diagnostic capability for patients with HF with preserved ejection fraction (HFpEF) when compared with control subjects. Higher plasma GDF15 concentrations were found in patients with HFrEF relative to patients with HF with midrange ejection fraction (HFmrEF) [130]. Another recent study sought to assess whether the prognostic value of GDF15 is superior to that of NT-proBNP in patients with HFmrEF or HFpEF. GDF15 was superior to NT-proBNP for assessing prognosis in patients with HFpEF and HFmrEF. GDF15 has emerged as a strong, independent biomarker for identifying HFmrEF and HFpEF patients with worse prognosis [131].

Concerning the question of whether GDF15 has an effect on ECs in vascular diseases, various studies in humans suggest a positive correlation between GDF15 serum levels, variations in the vascular properties of the lungs, and mean pulmonary artery pressure (PAH). High levels of GDF15 have been observed in certain pulmonary diseases, including chronic obstructive pulmonary disease (COPD) and PAH [132]. In the vasculature of the lungs, EC expression of the GDF15 gene is strongly upregulated in response to PAH. It has been demonstrated that pulmonary injury during PAH was associated with an increase in oxidative stress and inflammation. Chronic oxidation contributes to lung damage and disease progression. In human lung tissue from patients with idiopathic PAH, superoxide dismutase activity was reduced and the expression of oxidant stress markers was elevated [133,134].

As we reported previously, GDF15 can regulate vascular contraction and relaxation responses through an endothelial process implicating an increase in the release of NO [64]. In return, GDF15 was not associated with significant hemodynamic effects in GDF15-knockout mice. The absence of a clear vascular phenotype of these mice may be in relationship with the fact that GDF15 expression is low in control animals and induced following “injury”. It is important to note that GDF15 is linked with many endogenous pathways and that in the endothelium a variety of constitutive and inducible mechanisms are involved in the reduction of injury and tissue repair [135].

The process of vascularization is imperative during tissue development and regeneration, while angiogenesis is a deleterious process during tumorigenesis; angiogenesis being stimulated to supply oxygen and nutrients. GDF15 is highly associated with malignant human cancers and it has been suggested that it is involved in tumor angiogenesis. GDF15 secreted from cancer cells stimulates endothelial cell proliferation by enhancing AP-1- and E2F-dependent expression of G(1) cyclins via the PI3K/Akt signaling pathway, increasing angiogenesis [136]. Similar to other angiogenic factors, GDF15 has pro-angiogenic effects. Studies show that GDF15 promotes angiogenesis in hypoxic human umbilical vein endothelial cells (HUVECs) and that HIF-1α activation and signaling might be important mediators responsible for the GDF15-induced angiogenic response [82].

**Figure 3:**
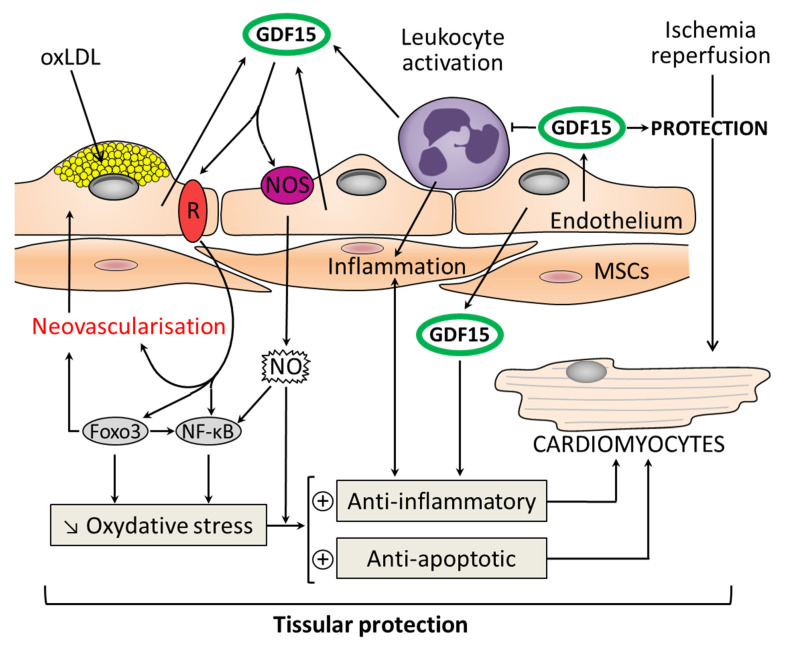
Molecular pathways for the action of GDF15 in myocardial protection as a result of its antioxidant, anti-inflammatory, and antiapoptotic properties. GDF15 has a local cardioprotective role due to its autocrine/paracrine functions. GDF15 expression is highly induced in cardiomyocytes after ischemia/reperfusion. GDF15 is an inhibitor of leukocyte activation and recruitment by direct interference with chemokine signaling and integrin activation. GDF15 activates nitric oxide: NOS/NO/nuclear factor κB (NFκB) pathway and among the forkhead box (FOX) transcription factors: Foxo3. This induces an antioxidant action and promotes neovascularization. The development of atherosclerotic plaques is driven by endothelial dysfunction, oxidized low-density lipoprotein (oxLDL) in association with recruitment of monocytes. GDF15 is highly expressed in plaques. GDF15 protects the vessel against injury and oxidative stress thanks to its anti-inflammatory and antiapoptotic properties (+: activation; ↓: decrease).

GDF15 production may rise in response to tissue injury in association with inflammation. GDF15 is the first anti-inflammatory cytokine to be shown to directly interfere with chemokine-triggered inside-out signaling, leading to leukocyte integrin activation (Figure 3). GDF15 is an inhibitor of leukocyte recruitment by direct interference with chemokine signaling and integrin activation in inflamed tissue [137]. The receptor responsible for this GDF15-triggered anti-inflammatory mechanism on myeloid cells has been identified. In inflamed tissue, GDF15 reduces chemokine-triggered leukocyte integrin activation (Figure 3) and neutrophil recruitment by interacting with a receptor-pair consisting of activin receptor-like kinase 5 (ALK-5) and TGF-β receptor II (TGF-βRII) [138].

In the field of medicine, sepsis remains a major problem facing critical care practitioners. Severe infections are among the leading causes of morbidity and mortality in intensive care. The pathophysiology of these septic states is complex, involving many mediators and leading to the activation of the inflammatory cascade. The inflammatory response to acute infection involves the activation of various cells such as leukocytes and other inflammatory cells (Figure 3) leading to a massive production of ROS and the NO pathway [139,140]. The anti-inflammatory role of GDF15 was highlighted in an experimental sepsis model in GDF15 knockout mice. These mice had increased inflammatory responses to lipopolysaccharide (LPS) and increased expression of monocyte chemoattractant protein (MCP)-1, IL-6, and TNF-α in both cardiac and renal tissues, leading to functional problems. The authors demonstrated the induction of GDF15 during sepsis and its tissue-protective role, defining GDF15 as an “inflammation-induced central mediator of tissue tolerance” [122]. Finally, GDF15 possesses immune-regulatory functions that have the potential to improve immunotherapies [141].

## 8. Perspectives for Clinical Applications of GDF15 in Cardiovascular Domain

Atherosclerosis is the main reason for morbidity and death caused by cardiovascular disease, leading to approximately 20% of all deaths worldwide. The development of atherosclerotic plaques is driven by endothelial dysfunction, specifically the deposition of oxidized low-density lipoprotein (oxLDL) in the subendothelial space in association with recruitment of inflammatory monocytes in the vessel wall. In addition, EC dysfunction due to the overproduction of oxLDLs leads to the imbalanced activation of NOS, expressed constitutively by ECs: cNOS, thereby facilitating the activation of the inducible isoform of this enzyme (iNOS). The activity of iNOS enhances inflammatory processes and contributes to atherosclerosis progression. Moreover, the imbalanced modulation of the cNOS/iNOS relationship appears to play a key role in vascular dysfunction, affecting the production of MMPs and the stability of the atherosclerotic plaque [142].

GDF15 is expressed in atherosclerotic plaques. GDF15 inhibits ECs proliferation in vitro, while in vivo GDF15 appears to have anti-inflammatory and antiapoptotic properties that limit tissue injury. However, inconsistent reports show that a deficiency of GDF15 prevents atherosclerosis [143]. The associations between GDF15 gene polymorphisms and atherosclerosis-related disease, including coronary disorders, were evoked; in return the results of studies in this field were incoherent [144]. In various studies, GDF15 treatments have induced cardioprotective effects, likely due to GDF’s autocrine/paracrine role. In cardiomyocytes subject to ischemia/reperfusion, GDF15 expression is stimulated. It has been reported in clinical studies that circulating levels of GDF15 were increased in patients with an acute coronary syndrome, Moreover, patients with elevated levels of GDF15 (>1800 ng/L) had a high risk of death within one year [145,146].

In experimental models of ischemia, mRNA and protein levels of GDF15 were increased during the onset and development of no-reflow. A negative relationship was shown between GDF15 expression and the activity of myeloperoxidase (MPO): an indicator of neutrophil infiltration. GDF15 inhibits the inflammatory-like response and this action is in relationship with specific properties involving the neutrophil infiltration and trans-endothelial migration [137,147].

In other instances of protection, GDF15 showed an antiapoptotic effect against ischemia/reperfusion (I/R) (Figure 3) and reduced the size of MI. In a recent study using GDF15 transgenic mice in vivo and adenovirus GDF15 expression in vitro, it was reported that GDF15 had a protective effect on cold I/R in heart transplantation. GDF15 exerts a protective effect through interaction with NFκB signaling and among the forkhead box (FOX) transcription factor Foxo3 [148]. A link exists between Foxo3 and NFκB signaling, both of which play important antiapoptotic roles and influence oxidative stress. A crosstalk exists between Foxo3 and NFκB signaling, and Foxo3 acts as an antagonist of NFκB signaling [149].

Interestingly, GDF15 and BMP-2 show similarities in their primary structure. BMPs, which belong to the TGF-β family, participate in organ regeneration through autocrine and paracrine actions. BMP-2 activates ALK-2/3/6 and phosphorylates Smad1/5. Similarly, GDF15 activates type I receptors and Smad1/5 [150]. The tissue protection in myocardial injury is constantly associated with a reduction in the recruitment of inflammatory cells. In this context, GDF15 plays a major role. The functional and metabolic links between GDF15 and ECs remain to be firmly established by future studies.

Several studies have investigated the prognostic value of GDF15 in acute coronary syndrome (ACS) and other heart diseases. Well known studies such as the Framingham Heart, MERLIN-TIMI, and PLATO studies are based on the acute phase of ACS. In the Framingham Heart Study, to determine the prognostic value of three biomarkers induced by cardiovascular stress, soluble ST2, GDF15, and high-sensitivity troponin I (cTnT-hs) were measured in 3428 participants. During a mean follow-up of 11.3 years, there were 488 deaths, 336 major CV events, 162 HF events, and 142 CAD. In multivariable-adjusted models, the three biomarkers were associated with each end point (*p* < 0.001) except coronary events. Concentrations of GDF15 were strongly associated with the risk of death and HF [151].

The MERLIN-TIMI study focused on the prognostic value of GDF15 in a population of 4330 non-ST-segment elevation (NSTE) myocardial infarction (NSTEMI) patients. There was an interaction between the TIMI risk score and GDF15. GDF15 was positively and independently associated with CV death and HF [152].

The PLATO trial evaluated 5174 NSTE-ACS patients who underwent initial angiography and revascularization along with measurement of serum cTnT-hs, NT-proBNP, and GDF15. The extent of CAD at coronary angiography and the values of NT-proBNP and GDF15 independently improved the prediction of CVD or MI. These markers contributed to the prognosis of both CVD and MI in these patients [152]. Increased concentrations of GDF15 were observed in patients at increased risk of adverse left ventricular remodeling after ACS [62]. In fully-adjusted models, GDF15 predicted death from heart disease [153].

In our own laboratory, we demonstrated that during cardiac surgery associated with cardiopulmonary bypass (CPB), circulating GDF15 levels increased and were associated with markers of cardiac injury and renal dysfunction [154]. These results were the first descriptions for the time course of GDF15 during cardiac surgery; a clinical state situation associated with CPB and an acute inflammatory response. In patients with no history of atrial fibrillation, a low plasma level of GDF15 before coronary artery bypass graft surgery was a strong independent predictor of postoperative atrial fibrillation (POAF) [155,156]. It has also been suggested that GDF15 could be used as a prognostic tool for stroke risk in patients with AF. In the ARISTOTLE trial, 18,201 patients with AF and anticoagulation treatment (apixaban or warfarin) were enrolled. In this trial, independent of age, GDF15 levels were higher in patients with AF than in healthy individuals [157].

Recent results obtained in our laboratory suggest that GDF15 could be an integrative biomarker for severe in-hospital HF (defined as Killip class > 2) in patients with acute myocardial infarction (AMI). Serum levels of GDF15 were obtained from blood samples taken on admission. Among the 284 AMI patients, the median age was 67 years, and 27% were women. GDF15 levels were strongly correlated with age and positively correlated with cardiovascular risk factors (hypertension and diabetes) and inflammation (C-reactive protein: CRP > 3 mg/L). When compared with patients without HF (274/284), GDF15 in patients with in-hospital HF (10/284) was more than two times higher. As a biomarker, GDF15 may provide an insight that complements the information obtained with traditional clinical or biochemical cardiovascular markers. In the long-term, increased GDF15 concentrations were shown to identify patients at risk of re-hospitalization for new or worsening HF, independently of other biomarkers such as high-sensitivity CRP and NT-proBNP [158]. Findings also suggested that the secreted protein FSTL-1 could be an upstream inducer of GDF15 production and an independent prognostic biomarker in ACSs [159]. Finally, circulating levels of GDF15 reflect acute and chronic cellular injuries and may, therefore, prove to be a highly useful biomarker [160].

## 9. GDF15 as an Emerging Biomarker Reflecting Mitochondrial Function

A biomarker is defined as a characteristic that is objectively measured and evaluated as an indicator of normal biological processes, pathogenic processes, or pharmacologic responses to a therapeutic intervention. They are by definition objective and quantifiable characteristics of biological processes. However, their relevance and validity must be identified beforehand [161,162]. Inflammation-related markers are prominent among validated and currently used biomarkers, which include GDF15. Associations have been reported between GDF15 and vascular and cardiac conditions.

New data in COVID-19 patients has shown that GDF15 presents a strong association with levels of soluble angiotensin-converting enzyme 2 (sACE2). ACE2, a homologue of ACE, degrades angiotensin (Ang-I) and Ang-II to produce Ang-(1–9) and Ang-(1–7), which have vasodilatory properties because they decrease the levels of Ang-II and increase the levels of Ang-(1–7) [163]. New investigations have demonstrated that the expression levels of ACE2, encoding angiotensin-converting enzyme 2, which is a receptor used by SARS-CoV-2 to infect cells, were highest in the heart in pericytes, followed by fibroblasts, and lastly in cardiomyocytes.

The COVID-19 pandemic is caused by the SARS-CoV-2 virus, which enters human cells using angiotensin-converting enzyme 2 (ACE2) as a cell surface receptor. Levels of GDF15, which are associated both with ACE2 levels and a higher risk of mortality, might be used as a biomarker to better identify the risk of severe COVID-19 infection [164]. Inflammation and GDF15 have already been associated, and higher levels of inflammatory blood biomarkers, such as CRP, ferritin, and D-dimers, have been reported as predictors of poor outcomes in COVID-19 patients [165]. Other recently-published results indicate that circulating GDF15 levels are elevated in patients hospitalized with COVID-19, and higher concentrations are associated with SARS-CoV-2 viremia, hypoxemia, and worse outcomes. The prognostic importance of GDF15 was superior to established inflammatory biomarkers [166]. Therefore, GDF15 has a role in inflammation-driven states and is a biomarker for sepsis. This concept is also demonstrated in patients with hemorrhagic shock and encephalopathy syndrome (HSES). The pathophysiology of these diseases, which have high early mortality rates, has been hypothesized to involve a ‘cytokine storm’. Serum levels of multiple cytokines, chemokines, and GDF15 were evaluated: the highest levels of cytokines, chemokines, and GDF15 were observed in the first 24 h, and decreased thereafter. The GDF15 level was markedly high, signaling the inflammation pathway [167].

In conclusion, plasma biomarkers that reflect molecular states of the cardiovascular system are central for clinical decision making. Routinely used plasma biomarkers include troponins, natriuretic peptides, and lipoprotein particles. However, because GDF15 protein concentrations can be easily determined in circulation, many studies have identified it as an important plasma biomarker that correlates with various diseases. As mentioned above, GDF15 is a useful diagnostic marker for mitochondrial diseases, which are inherited disorders caused by mitochondrial or nuclear genomic mutations, leading to impaired energy production. Considering that the primary cause of mitochondrial diseases is mitochondrial dysfunction, the level of GDF15 in the blood might reflect mitochondrial function in patients, and could, thus, be a suitable marker for mitochondrial dysfunction.

## 10. Concluding Remarks and Future Perspectives

GDF15 is emerging as a relevant contributor to energy homeostasis and it appears to be a biomarker of various cardiovascular and metabolic diseases. By modifying the amount of GDF15–GFRAL–RET signaling, a new class of GDF15–GFRAL–RET-based drugs is emerging such as new medications to treat neuropathic pain [168]. More recently, XIB4035, a nonpeptidyl agonist of GDNF receptor α1 (GFRα1), was found to be an active treatment for small-fiber neuropathy (SFN) [169].

Obesity and its related metabolic dysregulation are established risk factors for many conditions such as cardiovascular disease and cancer. However, the biological mechanisms underlying this relationship are still not fully understood. Obesity is characterized by low-grade systemic or chronic inflammation that is responsible for the release of endogenous factors such as cytokines, adipokines, and chemokines [170]. As previously stated, the production of GDF15 is part of the body’s response to tissue injury. Currently, inflammation is recognized as a set of complex changing responses to tissue injury caused by trauma, infection, or toxic compounds. GDF15 is the first anti-inflammatory cytokine proven to directly interfere with chemokine-triggered, inside-out signaling, leading to leukocyte integrin activation. As described, GDF15 is an inhibitor of polymorphonuclear leukocyte recruitment by direct interference with chemokine signaling and integrin activation in inflamed tissue [137]. Taken together, the overall results support the potential use of GDF15 as a novel therapeutic target (1) for preventing and treating obesity by modulating metabolic activity (2) for promoting adaptive angiogenesis in the heart, and (3) for promoting cardiac regeneration. Clinical trials attempting to evaluate these associations are currently ongoing.
